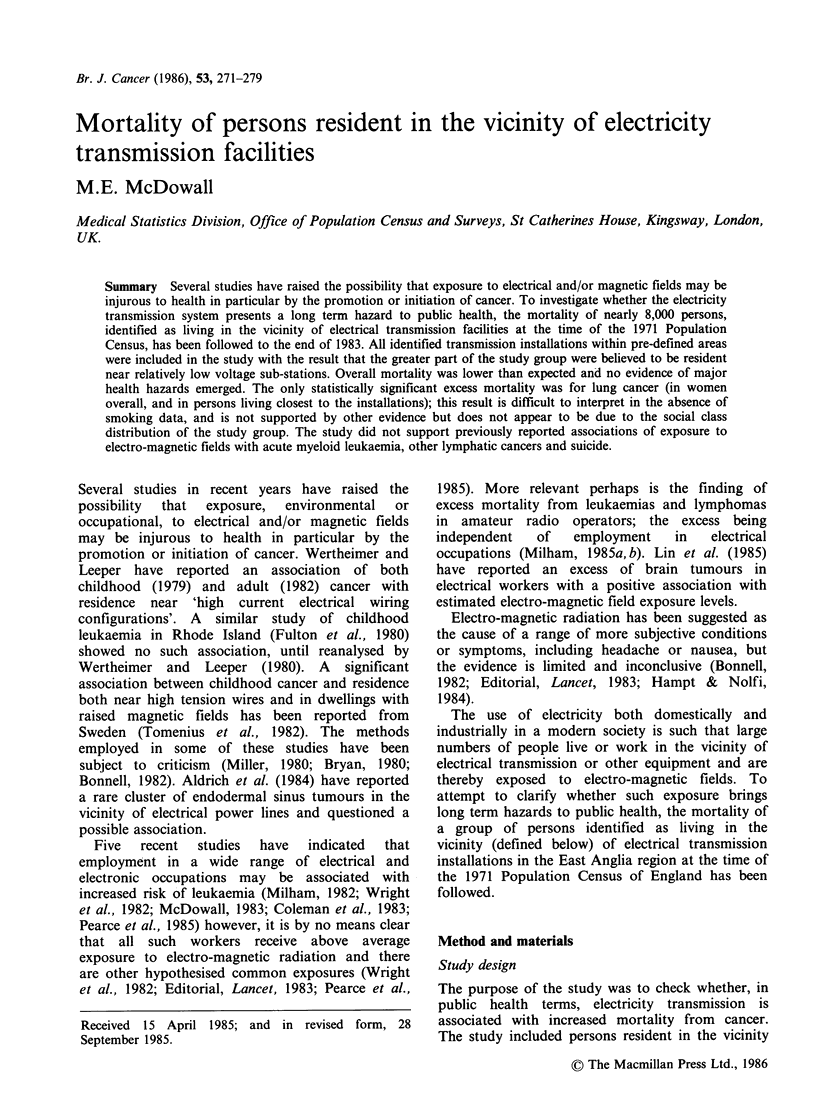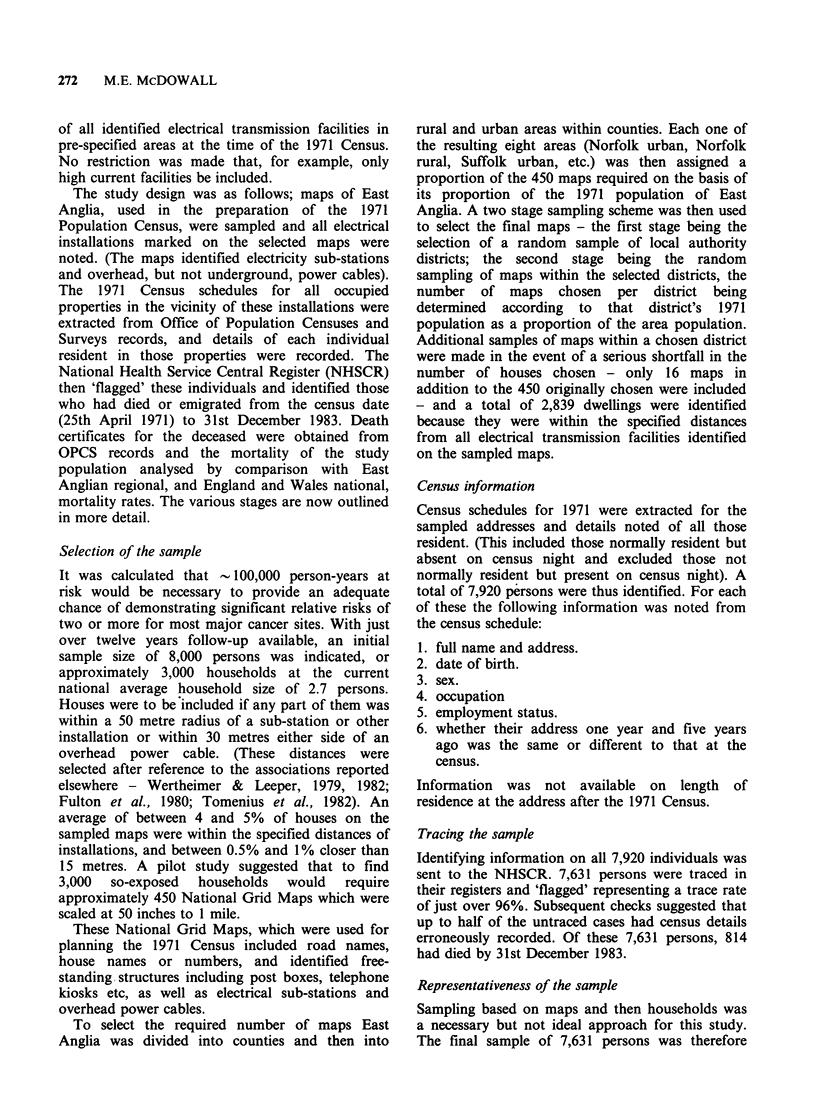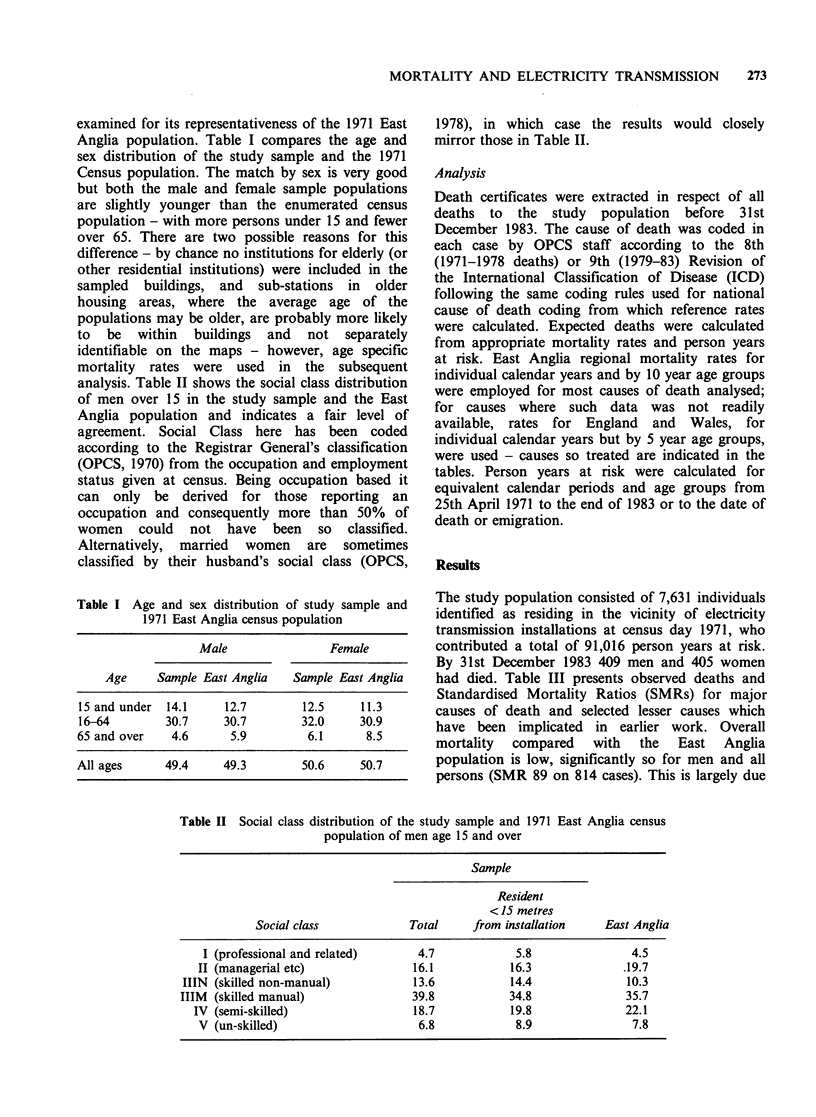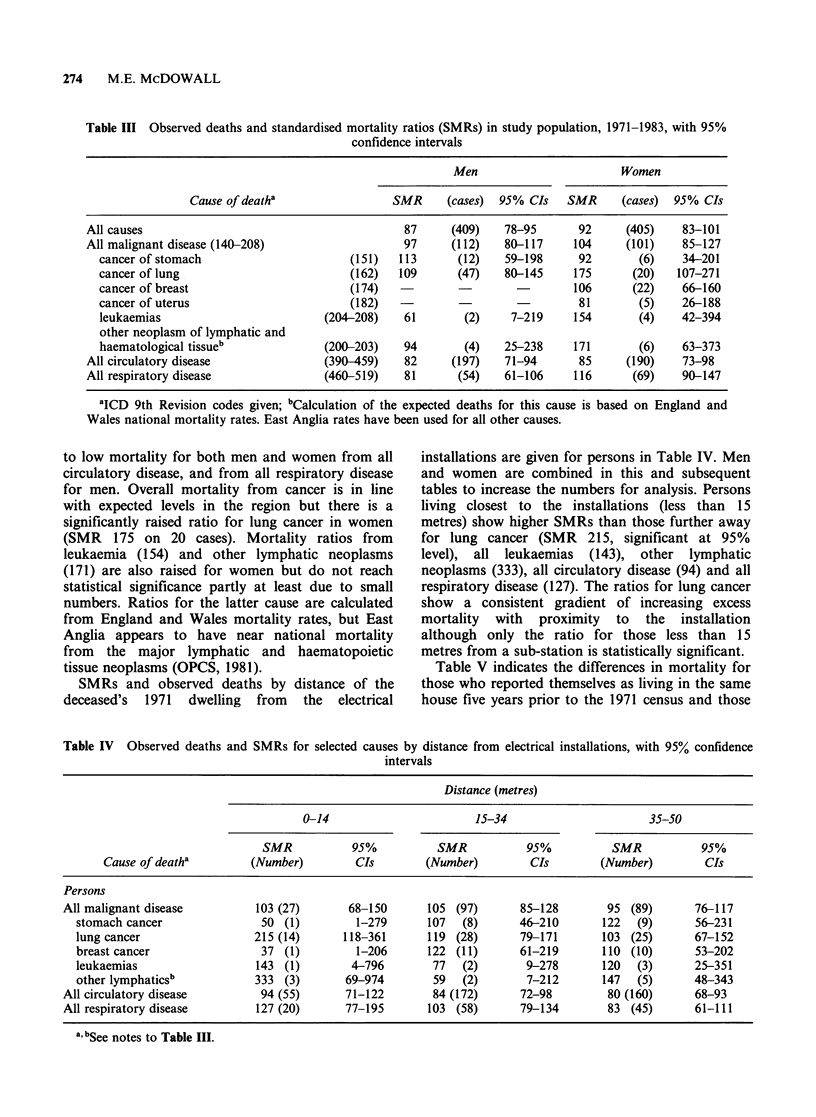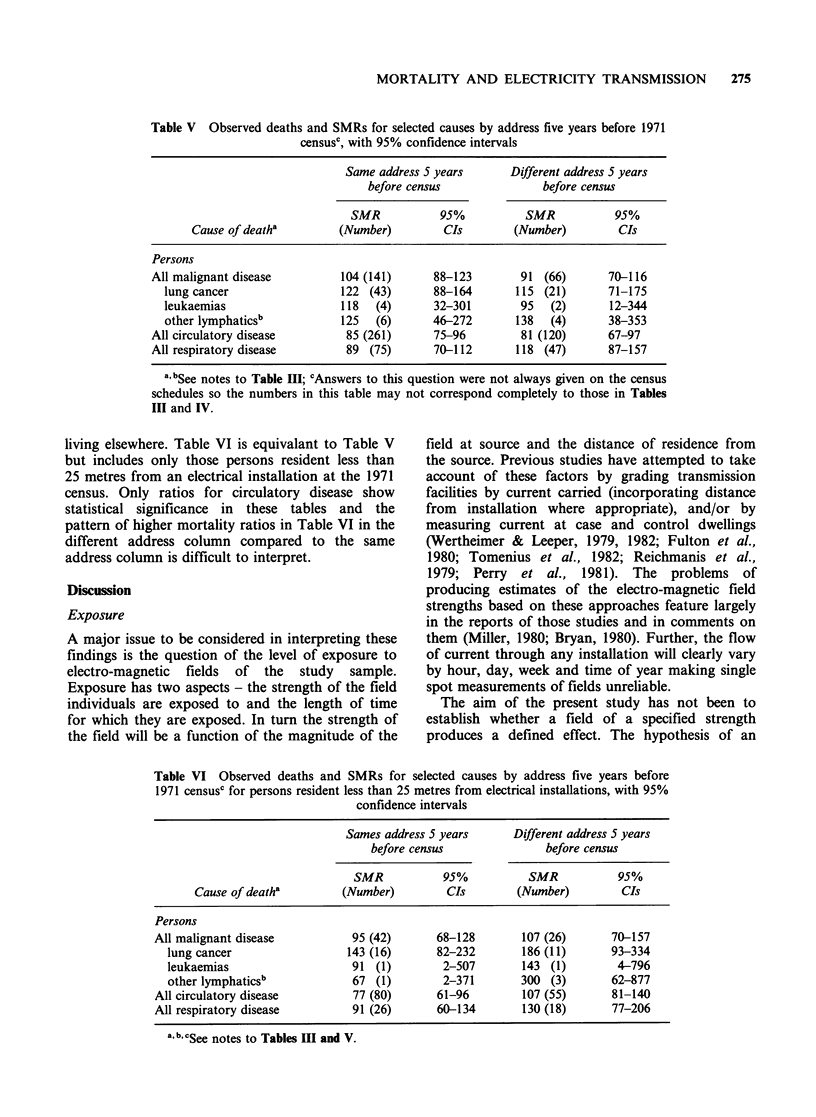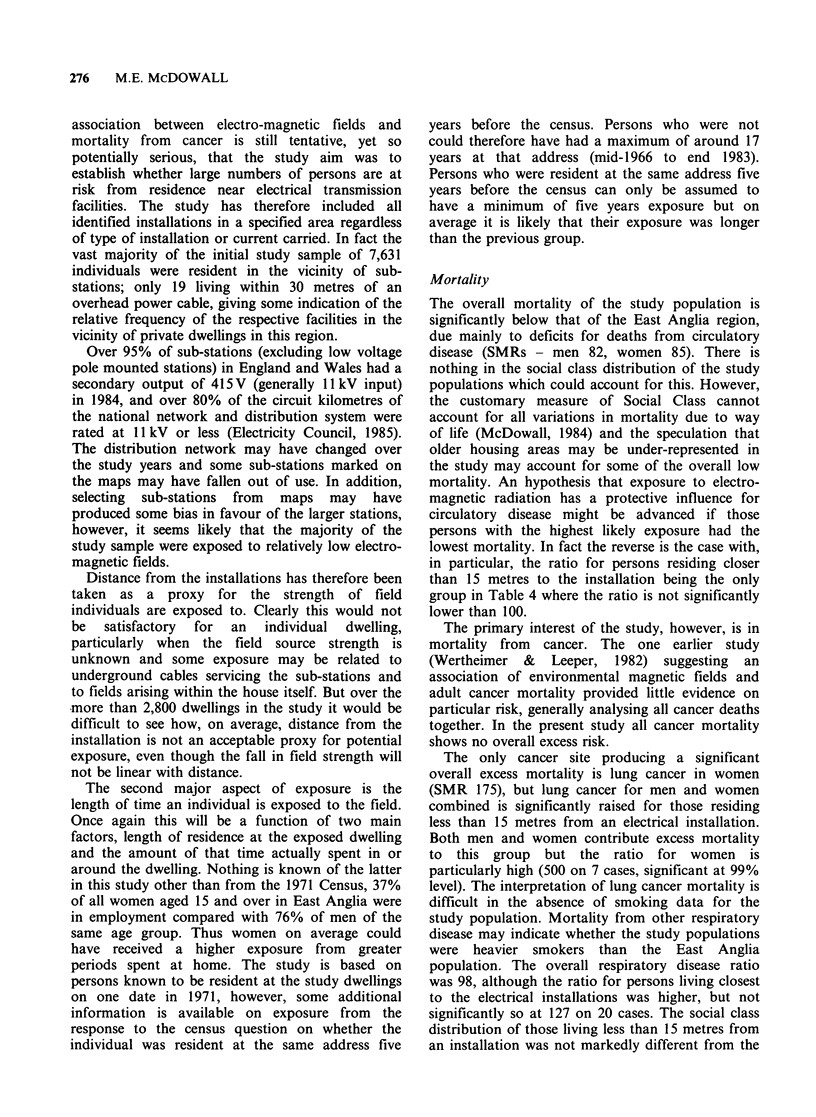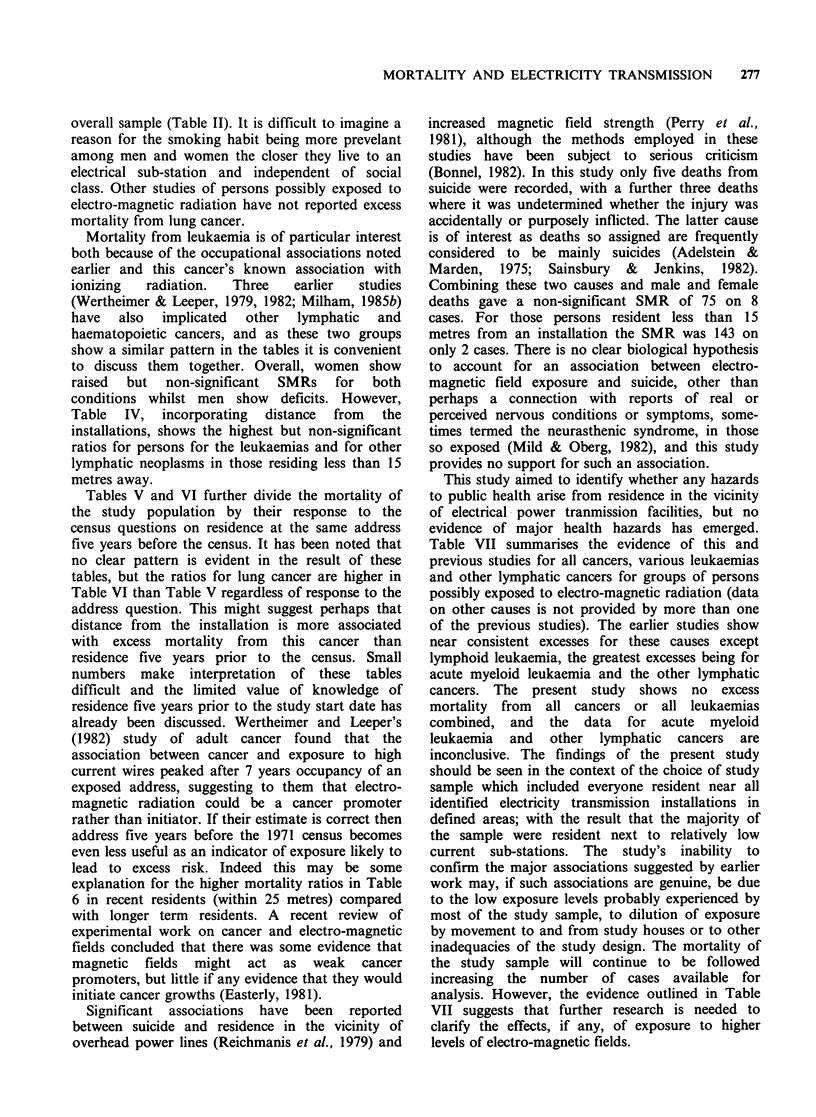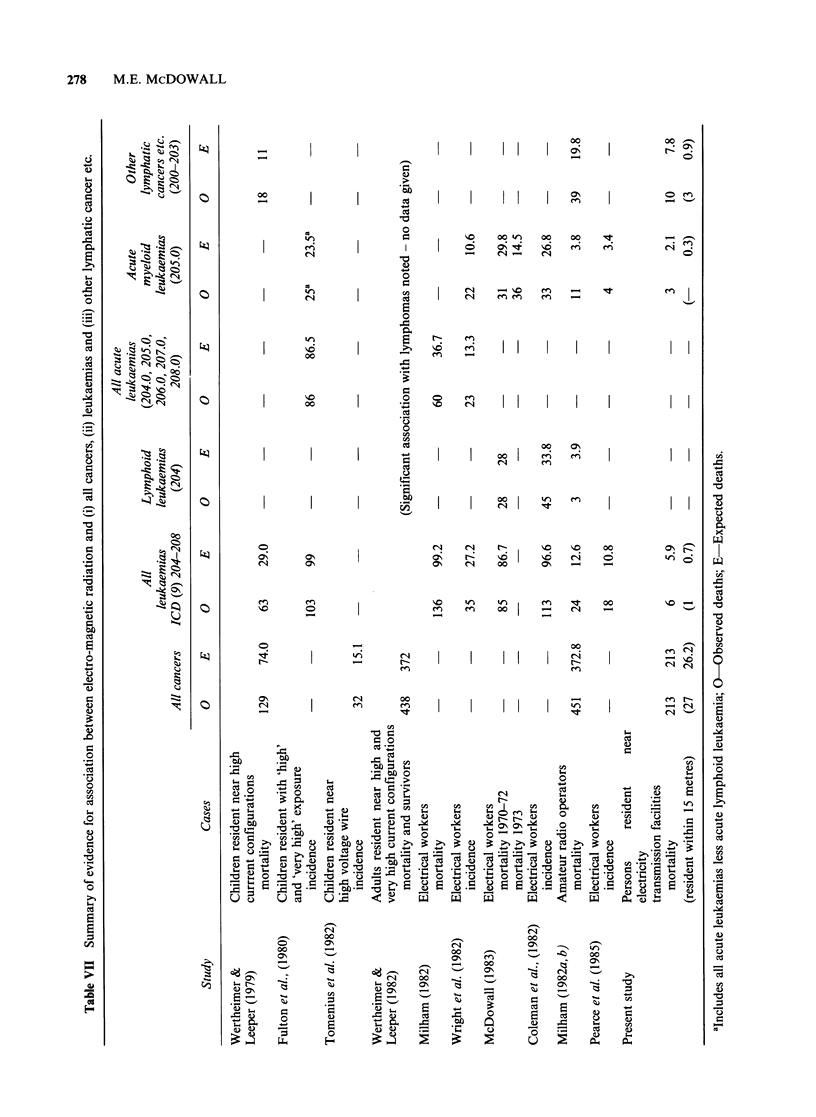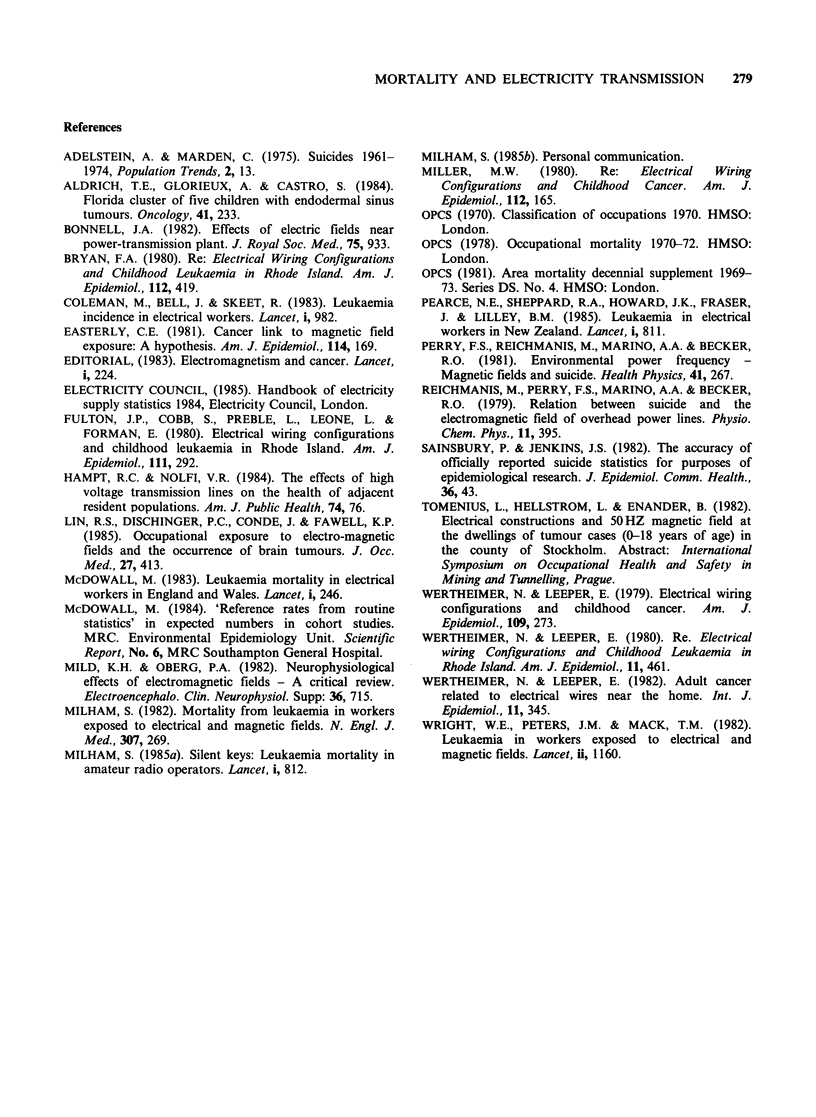# Mortality of persons resident in the vicinity of electricity transmission facilities.

**DOI:** 10.1038/bjc.1986.45

**Published:** 1986-02

**Authors:** M. E. McDowall

## Abstract

Several studies have raised the possibility that exposure to electrical and/or magnetic fields may be injurious to health in particular by the promotion or initiation of cancer. To investigate whether the electricity transmission system presents a long term hazard to public health, the mortality of nearly 8,000 persons, identified as living in the vicinity of electrical transmission facilities at the time of the 1971 Population Census, has been followed to the end of 1983. All identified transmission installations within pre-defined areas were included in the study with the result that the greater part of the study group were believed to be resident near relatively low voltage sub-stations. Overall mortality was lower than expected and no evidence of major health hazards emerged. The only statistically significant excess mortality was for lung cancer (in women overall, and in persons living closest to the installations); this result is difficult to interpret in the absence of smoking data, and is not supported by other evidence but does not appear to be due to the social class distribution of the study group. The study did not support previously reported associations of exposure to electro-magnetic fields with acute myeloid leukaemia, other lymphatic cancers and suicide.


*Br. J. Cancer* (1986), **53**, 271–279

### Mortality of persons resident in the vicinity of electricity transmission facilities

M.E. McDowall

*Medical Statistics Division, Office of Population Census and Surveys, St Catherines House, Kingsway, London, UK.*

**Summary** Several studies have raised the possibility that exposure to electrical and/or magnetic fields may be injurous to health in particular by the promotion or initiation of cancer. To investigate whether the electricity transmission system presents a long term hazard to public health, the mortality of nearly 8,000 persons, identified as living in the vicinity of electrical transmission facilities at the time of the 1971 Population Census, has been followed to the end of 1983. All identified transmission installations within pre-defined areas were included in the study with the result that the greater part of the study group were believed to be resident near relatively low voltage sub-stations. Overall mortality was lower than expected and no evidence of major health hazards emerged. The only statistically significant excess mortality was for lung cancer (in women overall, and in persons living closest to the installations); this result is difficult to interpret in the absence of smoking data, and is not supported by other evidence but does not appear to be due to the social class distribution of the study group. The study did not support previously reported associations of exposure to electro-magnetic fields with acute myeloid leukaemia, other lymphatic cancers and suicide.

Several studies in recent years have raised the possibility that exposure, environmental or occupational, to electrical and/or magnetic fields may be injurous to health in particular by the promotion or initiation of cancer. Wertheimer and Leeper have reported an association of both childhood (1979) and adult (1982) cancer with residence near 'high current electrical wiring configurations'. A similar study of childhood leukaemia in Rhode Island (Fulton *et al.*, 1980) showed no such association, until reanalysed by Wertheimer and Leeper (1980). A significant association between childhood cancer and residence both near high tension wires and in dwellings with raised magnetic fields has been reported from Sweden (Tomenius *et al.*, 1982). The methods employed in some of these studies have been subject to criticism (Miller, 1980; Bryan, 1980; Bonnell, 1982). Aldrich *et al.* (1984) have reported a rare cluster of endodermal sinus tumours in the vicinity of electrical power lines and questioned a possible association.

Five recent studies have indicated that employment in a wide range of electrical and electronic occupations may be associated with increased risk of leukaemia (Milham, 1982; Wright *et al.*, 1982; McDowall, 1983; Coleman *et al.*, 1983; Pearce *et al.*, 1985) however, it is by no means clear that all such workers receive above average exposure to electro-magnetic radiation and there are other hypothesised common exposures (Wright *et al.*, 1982; Editorial, *Lancet*, 1983; Pearce *et al.*, 1985). More relevant perhaps is the finding of excess mortality from leukaemias and lymphomas in amateur radio operators; the excess being independent of employment in electrical occupations (Milham, 1985*a,b*). Lin *et al.* (1985) have reported an excess of brain tumours in electrical workers with a positive association with estimated electro-magnetic field exposure levels.

Electro-magnetic radiation has been suggested as the cause of a range of more subjective conditions or symptoms, including headache or nausea, but the evidence is limited and inconclusive (Bonnell, 1982; Editorial, *Lancet*, 1983; Hampt & Nolfi, 1984).

The use of electricity both domestically and industrially in a modern society is such that large numbers of people live or work in the vicinity of electrical transmission or other equipment and are thereby exposed to electro-magnetic fields. To attempt to clarify whether such exposure brings long term hazards to public health, the mortality of a group of persons identified as living in the vicinity (defined below) of electrical transmission installations in the East Anglia region at the time of the 1971 Population Census of England has been followed.

### Method and materials

### *Study design*

The purpose of the study was to check whether, in public health terms, electricity transmission is associated with increased mortality from cancer. The study included persons resident in the vicinity

Received 15 April 1985; and in revised form, 28 September 1985.

© The Macmillan Press Ltd., 1986

of all identified electrical transmission facilities in pre-specified areas at the time of the 1971 Census. No restriction was made that, for example, only high current facilities be included.

The study design was as follows; maps of East Anglia, used in the preparation of the 1971 Population Census, were sampled and all electrical installations marked on the selected maps were noted. (The maps identified electricity sub-stations and overhead, but not underground, power cables). The 1971 Census schedules for all occupied properties in the vicinity of these installations were extracted from Office of Population Censuses and Surveys records, and details of each individual resident in those properties were recorded. The National Health Service Central Register (NHSCR) then 'flagged' these individuals and identified those who had died or emigrated from the census date (25th April 1971) to 31st December 1983. Death certificates for the deceased were obtained from OPCS records and the mortality of the study population analysed by comparison with East Anglian regional, and England and Wales national, mortality rates. The various stages are now outlined in more detail.

*Selection of the sample*

It was calculated that ~100,000 person-years at risk would be necessary to provide an adequate chance of demonstrating significant relative risks of two or more for most major cancer sites. With just over twelve years follow-up available, an initial sample size of 8,000 persons was indicated, or approximately 3,000 households at the current national average household size of 2.7 persons. Houses were to be included if any part of them was within a 50 metre radius of a sub-station or other installation or within 30 metres either side of an overhead power cable. (These distances were selected after reference to the associations reported elsewhere – Wertheimer & Leeper, 1979, 1982; Fulton *et al.*, 1980; Tomenius *et al.*, 1982). An average of between 4 and 5% of houses on the sampled maps were within the specified distances of installations, and between 0.5% and 1% closer than 15 metres. A pilot study suggested that to find 3,000 so-exposed households would require approximately 450 National Grid Maps which were scaled at 50 inches to 1 mile.

These National Grid Maps, which were used for planning the 1971 Census included road names, house names or numbers, and identified free-standing structures including post boxes, telephone kiosks etc, as well as electrical sub-stations and overhead power cables.

To select the required number of maps East Anglia was divided into counties and then into rural and urban areas within counties. Each one of the resulting eight areas (Norfolk urban, Norfolk rural, Suffolk urban, etc.) was then assigned a proportion of the 450 maps required on the basis of its proportion of the 1971 population of East Anglia. A two stage sampling scheme was then used to select the final maps – the first stage being the selection of a random sample of local authority districts; the second stage being the random sampling of maps within the selected districts, the number of maps chosen per district being determined according to that district's 1971 population as a proportion of the area population. Additional samples of maps within a chosen district were made in the event of a serious shortfall in the number of houses chosen – only 16 maps in addition to the 450 originally chosen were included – and a total of 2,839 dwellings were identified because they were within the specified distances from all electrical transmission facilities identified on the sampled maps.

*Census information*

Census schedules for 1971 were extracted for the sampled addresses and details noted of all those resident. (This included those normally resident but absent on census night and excluded those not normally resident but present on census night). A total of 7,920 persons were thus identified. For each of these the following information was noted from the census schedule:

1. full name and address.

2. date of birth.

3. sex.

4. occupation

5. employment status.

6. whether their address one year and five years ago was the same or different to that at the census.

Information was not available on length of residence at the address after the 1971 Census.

*Tracing the sample*

Identifying information on all 7,920 individuals was sent to the NHSCR. 7,631 persons were traced in their registers and 'flagged' representing a trace rate of just over 96%. Subsequent checks suggested that up to half of the untraced cases had census details erroneously recorded. Of these 7,631 persons, 814 had died by 31st December 1983.

*Representativeness of the sample*

Sampling based on maps and then households was a necessary but not ideal approach for this study. The final sample of 7,631 persons was therefore examined for its representativeness of the 1971 East Anglia population. Table I compares the age and sex distribution of the study sample and the 1971 Census population. The match by sex is very good but both the male and female sample populations are slightly younger than the enumerated census population – with more persons under 15 and fewer over 65. There are two possible reasons for this difference – by chance no institutions for elderly (or other residential institutions) were included in the sampled buildings, and sub-stations in older housing areas, where the average age of the populations may be older, are probably more likely to be within buildings and not separately identifiable on the maps – however, age specific mortality rates were used in the subsequent analysis. Table II shows the social class distribution of men over 15 in the study sample and the East Anglia population and indicates a fair level of agreement. Social Class here has been coded according to the Registrar General's classification (OPCS, 1970) from the occupation and employment status given at census. Being occupation based it can only be derived for those reporting an occupation and consequently more than 50% of women could not have been so classified. Alternatively, married women are sometimes classified by their husband's social class (OPCS, 1978), in which case the results would closely mirror those in Table II.

**Table I** Age and sex distribution of study sample and 1971 East Anglia census population

| | *Male* | | *Female* | |
|---|---|---|---|---|
| *Age* | *Sample* | *East Anglia* | *Sample* | *East Anglia* |
| 15 and under | 14.1 | 12.7 | 12.5 | 11.3 |
| 16–64 | 30.7 | 30.7 | 32.0 | 30.9 |
| 65 and over | 4.6 | 5.9 | 6.1 | 8.5 |
| All ages | 49.4 | 49.3 | 50.6 | 50.7 |

*Analysis*

Death certificates were extracted in respect of all deaths to the study population before 31st December 1983. The cause of death was coded in each case by OPCS staff according to the 8th (1971–1978 deaths) or 9th (1979–83) Revision of the International Classification of Disease (ICD) following the same coding rules used for national cause of death coding from which reference rates were calculated. Expected deaths were calculated from appropriate mortality rates and person years at risk. East Anglia regional mortality rates for individual calendar years and by 10 year age groups were employed for most causes of death analysed; for causes where such data was not readily available, rates for England and Wales, for individual calendar years but by 5 year age groups, were used – causes so treated are indicated in the tables. Person years at risk were calculated for equivalent calendar periods and age groups from 25th April 1971 to the end of 1983 or to the date of death or emigration.

### Results

The study population consisted of 7,631 individuals identified as residing in the vicinity of electricity transmission installations at census day 1971, who contributed a total of 91,016 person years at risk. By 31st December 1983 409 men and 405 women had died. Table III presents observed deaths and Standardised Mortality Ratios (SMRs) for major causes of death and selected lesser causes which have been implicated in earlier work. Overall mortality compared with the East Anglia population is low, significantly so for men and all persons (SMR 89 on 814 cases). This is largely due

**Table II** Social class distribution of the study sample and 1971 East Anglia census population of men age 15 and over

| | *Sample* | | |
|---|---|---|---|
| *Social class* | *Total* | *Resident <15 metres from installation* | *East Anglia* |
| I (professional and related) | 4.7 | 5.8 | 4.5 |
| II (managerial etc) | 16.1 | 16.3 | .19.7 |
| IIIN (skilled non-manual) | 13.6 | 14.4 | 10.3 |
| IIIM (skilled manual) | 39.8 | 34.8 | 35.7 |
| IV (semi-skilled) | 18.7 | 19.8 | 22.1 |
| V (un-skilled) | 6.8 | 8.9 | 7.8 |

**Table III** Observed deaths and standardised mortality ratios (SMRs) in study population, 1971–1983, with 95% confidence intervals

| | | Men | | | Women | | |
|---|---|---|---|---|---|---|---|
| *Cause of death*[a] | | *SMR* | *(cases)* | *95% CIs* | *SMR* | *(cases)* | *95% CIs* |
| All causes | | 87 | (409) | 78–95 | 92 | (405) | 83–101 |
| All malignant disease (140–208) | | 97 | (112) | 80–117 | 104 | (101) | 85–127 |
| cancer of stomach | (151) | 113 | (12) | 59–198 | 92 | (6) | 34–201 |
| cancer of lung | (162) | 109 | (47) | 80–145 | 175 | (20) | 107–271 |
| cancer of breast | (174) | — | — | — | 106 | (22) | 66–160 |
| cancer of uterus | (182) | — | — | — | 81 | (5) | 26–188 |
| leukaemias | (204–208) | 61 | (2) | 7–219 | 154 | (4) | 42–394 |
| other neoplasm of lymphatic and haematological tissue[b] | (200–203) | 94 | (4) | 25–238 | 171 | (6) | 63–373 |
| All circulatory disease | (390–459) | 82 | (197) | 71–94 | 85 | (190) | 73–98 |
| All respiratory disease | (460–519) | 81 | (54) | 61–106 | 116 | (69) | 90–147 |

[a]ICD 9th Revision codes given; [b]Calculation of the expected deaths for this cause is based on England and Wales national mortality rates. East Anglia rates have been used for all other causes.

to low mortality for both men and women from all circulatory disease, and from all respiratory disease for men. Overall mortality from cancer is in line with expected levels in the region but there is a significantly raised ratio for lung cancer in women (SMR 175 on 20 cases). Mortality ratios from leukaemia (154) and other lymphatic neoplasms (171) are also raised for women but do not reach statistical significance partly at least due to small numbers. Ratios for the latter cause are calculated from England and Wales mortality rates, but East Anglia appears to have near national mortality from the major lymphatic and haematopoietic tissue neoplasms (OPCS, 1981).

SMRs and observed deaths by distance of the deceased's 1971 dwelling from the electrical installations are given for persons in Table IV. Men and women are combined in this and subsequent tables to increase the numbers for analysis. Persons living closest to the installations (less than 15 metres) show higher SMRs than those further away for lung cancer (SMR 215, significant at 95% level), all leukaemias (143), other lymphatic neoplasms (333), all circulatory disease (94) and all respiratory disease (127). The ratios for lung cancer show a consistent gradient of increasing excess mortality with proximity to the installation although only the ratio for those less than 15 metres from a sub-station is statistically significant.

Table V indicates the differences in mortality for those who reported themselves as living in the same house five years prior to the 1971 census and those

**Table IV** Observed deaths and SMRs for selected causes by distance from electrical installations, with 95% confidence intervals

| | Distance (metres) | | | | | |
|---|---|---|---|---|---|---|
| | 0–14 | | 15–34 | | 35–50 | |
| *Cause of death*[a] | *SMR (Number)* | *95% CIs* | *SMR (Number)* | *95% CIs* | *SMR (Number)* | *95% CIs* |
| *Persons* | | | | | | |
| All malignant disease | 103 (27) | 68–150 | 105 (97) | 85–128 | 95 (89) | 76–117 |
| stomach cancer | 50 (1) | 1–279 | 107 (8) | 46–210 | 122 (9) | 56–231 |
| lung cancer | 215 (14) | 118–361 | 119 (28) | 79–171 | 103 (25) | 67–152 |
| breast cancer | 37 (1) | 1–206 | 122 (11) | 61–219 | 110 (10) | 53–202 |
| leukaemias | 143 (1) | 4–796 | 77 (2) | 9–278 | 120 (3) | 25–351 |
| other lymphatics[b] | 333 (3) | 69–974 | 59 (2) | 7–212 | 147 (5) | 48–343 |
| All circulatory disease | 94 (55) | 71–122 | 84 (172) | 72–98 | 80 (160) | 68–93 |
| All respiratory disease | 127 (20) | 77–195 | 103 (58) | 79–134 | 83 (45) | 61–111 |

[a, b]See notes to **Table III**.

**Table V** Observed deaths and SMRs for selected causes by address five years before 1971 census[c], with 95% confidence intervals

| | *Same address 5 years before census* | | *Different address 5 years before census* | |
|---|---|---|---|---|
| *Cause of death*[a] | *SMR (Number)* | *95% CIs* | *SMR (Number)* | *95% CIs* |
| *Persons* | | | | |
| All malignant disease | 104 (141) | 88–123 | 91 (66) | 70–116 |
| lung cancer | 122 (43) | 88–164 | 115 (21) | 71–175 |
| leukaemias | 118 (4) | 32–301 | 95 (2) | 12–344 |
| other lymphatics[b] | 125 (6) | 46–272 | 138 (4) | 38–353 |
| All circulatory disease | 85 (261) | 75–96 | 81 (120) | 67–97 |
| All respiratory disease | 89 (75) | 70–112 | 118 (47) | 87–157 |

[a, b]See notes to **Table III**; [c]Answers to this question were not always given on the census schedules so the numbers in this table may not correspond completely to those in **Tables III** and **IV**.

living elsewhere. Table VI is equivalant to Table V but includes only those persons resident less than 25 metres from an electrical installation at the 1971 census. Only ratios for circulatory disease show statistical significance in these tables and the pattern of higher mortality ratios in Table VI in the different address column compared to the same address column is difficult to interpret.

### Discussion

### *Exposure*

A major issue to be considered in interpreting these findings is the question of the level of exposure to electro-magnetic fields of the study sample. Exposure has two aspects – the strength of the field individuals are exposed to and the length of time for which they are exposed. In turn the strength of the field will be a function of the magnitude of the field at source and the distance of residence from the source. Previous studies have attempted to take account of these factors by grading transmission facilities by current carried (incorporating distance from installation where appropriate), and/or by measuring current at case and control dwellings (Wertheimer & Leeper, 1979, 1982; Fulton *et al.*, 1980; Tomenius *et al.*, 1982; Reichmanis *et al.*, 1979; Perry *et al.*, 1981). The problems of producing estimates of the electro-magnetic field strengths based on these approaches feature largely in the reports of those studies and in comments on them (Miller, 1980; Bryan, 1980). Further, the flow of current through any installation will clearly vary by hour, day, week and time of year making single spot measurements of fields unreliable.

The aim of the present study has not been to establish whether a field of a specified strength produces a defined effect. The hypothesis of an

**Table VI** Observed deaths and SMRs for selected causes by address five years before 1971 census[c] for persons resident less than 25 metres from electrical installations, with 95% confidence intervals

| | *Sames address 5 years before census* | | *Different address 5 years before census* | |
|---|---|---|---|---|
| *Cause of death*[a] | *SMR (Number)* | *95% CIs* | *SMR (Number)* | *95% CIs* |
| *Persons* | | | | |
| All malignant disease | 95 (42) | 68–128 | 107 (26) | 70–157 |
| lung cancer | 143 (16) | 82–232 | 186 (11) | 93–334 |
| leukaemias | 91 (1) | 2–507 | 143 (1) | 4–796 |
| other lymphatics[b] | 67 (1) | 2–371 | 300 (3) | 62–877 |
| All circulatory disease | 77 (80) | 61–96 | 107 (55) | 81–140 |
| All respiratory disease | 91 (26) | 60–134 | 130 (18) | 77–206 |

[a, b, c]See notes to **Tables III and V**.

association between electro-magnetic fields and mortality from cancer is still tentative, yet so potentially serious, that the study aim was to establish whether large numbers of persons are at risk from residence near electrical transmission facilities. The study has therefore included all identified installations in a specified area regardless of type of installation or current carried. In fact the vast majority of the initial study sample of 7,631 individuals were resident in the vicinity of sub-stations; only 19 living within 30 metres of an overhead power cable, giving some indication of the relative frequency of the respective facilities in the vicinity of private dwellings in this region.

Over 95% of sub-stations (excluding low voltage pole mounted stations) in England and Wales had a secondary output of 415 V (generally 11 kV input) in 1984, and over 80% of the circuit kilometres of the national network and distribution system were rated at 11 kV or less (Electricity Council, 1985). The distribution network may have changed over the study years and some sub-stations marked on the maps may have fallen out of use. In addition, selecting sub-stations from maps may have produced some bias in favour of the larger stations, however, it seems likely that the majority of the study sample were exposed to relatively low electro-magnetic fields.

Distance from the installations has therefore been taken as a proxy for the strength of field individuals are exposed to. Clearly this would not be satisfactory for an individual dwelling, particularly when the field source strength is unknown and some exposure may be related to underground cables servicing the sub-stations and to fields arising within the house itself. But over the more than 2,800 dwellings in the study it would be difficult to see how, on average, distance from the installation is not an acceptable proxy for potential exposure, even though the fall in field strength will not be linear with distance.

The second major aspect of exposure is the length of time an individual is exposed to the field. Once again this will be a function of two main factors, length of residence at the exposed dwelling and the amount of that time actually spent in or around the dwelling. Nothing is known of the latter in this study other than from the 1971 Census, 37% of all women aged 15 and over in East Anglia were in employment compared with 76% of men of the same age group. Thus women on average could have received a higher exposure from greater periods spent at home. The study is based on persons known to be resident at the study dwellings on one date in 1971, however, some additional information is available on exposure from the response to the census question on whether the individual was resident at the same address five years before the census. Persons who were not could therefore have had a maximum of around 17 years at that address (mid-1966 to end 1983). Persons who were resident at the same address five years before the census can only be assumed to have a minimum of five years exposure but on average it is likely that their exposure was longer than the previous group.

*Mortality*

The overall mortality of the study population is significantly below that of the East Anglia region, due mainly to deficits for deaths from circulatory disease (SMRs – men 82, women 85). There is nothing in the social class distribution of the study populations which could account for this. However, the customary measure of Social Class cannot account for all variations in mortality due to way of life (McDowall, 1984) and the speculation that older housing areas may be under-represented in the study may account for some of the overall low mortality. An hypothesis that exposure to electro-magnetic radiation has a protective influence for circulatory disease might be advanced if those persons with the highest likely exposure had the lowest mortality. In fact the reverse is the case with, in particular, the ratio for persons residing closer than 15 metres to the installation being the only group in Table 4 where the ratio is not significantly lower than 100.

The primary interest of the study, however, is in mortality from cancer. The one earlier study (Wertheimer & Leeper, 1982) suggesting an association of environmental magnetic fields and adult cancer mortality provided little evidence on particular risk, generally analysing all cancer deaths together. In the present study all cancer mortality shows no overall excess risk.

The only cancer site producing a significant overall excess mortality is lung cancer in women (SMR 175), but lung cancer for men and women combined is significantly raised for those residing less than 15 metres from an electrical installation. Both men and women contribute excess mortality to this group but the ratio for women is particularly high (500 on 7 cases, significant at 99% level). The interpretation of lung cancer mortality is difficult in the absence of smoking data for the study population. Mortality from other respiratory disease may indicate whether the study populations were heavier smokers than the East Anglia population. The overall respiratory disease ratio was 98, although the ratio for persons living closest to the electrical installations was higher, but not significantly so at 127 on 20 cases. The social class distribution of those living less than 15 metres from an installation was not markedly different from the overall sample (Table II). It is difficult to imagine a reason for the smoking habit being more prevelant among men and women the closer they live to an electrical sub-station and independent of social class. Other studies of persons possibly exposed to electro-magnetic radiation have not reported excess mortality from lung cancer.

Mortality from leukaemia is of particular interest both because of the occupational associations noted earlier and this cancer's known association with ionizing radiation. Three earlier studies (Wertheimer & Leeper, 1979, 1982; Milham, 1985*b*) have also implicated other lymphatic and haematopoietic cancers, and as these two groups show a similar pattern in the tables it is convenient to discuss them together. Overall, women show raised but non-significant SMRs for both conditions whilst men show deficits. However, Table IV, incorporating distance from the installations, shows the highest but non-significant ratios for persons for the leukaemias and for other lymphatic neoplasms in those residing less than 15 metres away.

Tables V and VI further divide the mortality of the study population by their response to the census questions on residence at the same address five years before the census. It has been noted that no clear pattern is evident in the result of these tables, but the ratios for lung cancer are higher in Table VI than Table V regardless of response to the address question. This might suggest perhaps that distance from the installation is more associated with excess mortality from this cancer than residence five years prior to the census. Small numbers make interpretation of these tables difficult and the limited value of knowledge of residence five years prior to the study start date has already been discussed. Wertheimer and Leeper's (1982) study of adult cancer found that the association between cancer and exposure to high current wires peaked after 7 years occupancy of an exposed address, suggesting to them that electro-magnetic radiation could be a cancer promoter rather than initiator. If their estimate is correct then address five years before the 1971 census becomes even less useful as an indicator of exposure likely to lead to excess risk. Indeed this may be some explanation for the higher mortality ratios in Table 6 in recent residents (within 25 metres) compared with longer term residents. A recent review of experimental work on cancer and electro-magnetic fields concluded that there was some evidence that magnetic fields might act as weak cancer promoters, but little if any evidence that they would initiate cancer growths (Easterly, 1981).

Significant associations have been reported between suicide and residence in the vicinity of overhead power lines (Reichmanis *et al.*, 1979) and increased magnetic field strength (Perry *et al.*, 1981), although the methods employed in these studies have been subject to serious criticism (Bonnel, 1982). In this study only five deaths from suicide were recorded, with a further three deaths where it was undetermined whether the injury was accidentally or purposely inflicted. The latter cause is of interest as deaths so assigned are frequently considered to be mainly suicides (Adelstein & Marden, 1975; Sainsbury & Jenkins, 1982). Combining these two causes and male and female deaths gave a non-significant SMR of 75 on 8 cases. For those persons resident less than 15 metres from an installation the SMR was 143 on only 2 cases. There is no clear biological hypothesis to account for an association between electro-magnetic field exposure and suicide, other than perhaps a connection with reports of real or perceived nervous conditions or symptoms, sometimes termed the neurasthenic syndrome, in those so exposed (Mild & Oberg, 1982), and this study provides no support for such an association.

This study aimed to identify whether any hazards to public health arise from residence in the vicinity of electrical power tranmission facilities, but no evidence of major health hazards has emerged. Table VII summarises the evidence of this and previous studies for all cancers, various leukaemias and other lymphatic cancers for groups of persons possibly exposed to electro-magnetic radiation (data on other causes is not provided by more than one of the previous studies). The earlier studies show near consistent excesses for these causes except lymphoid leukaemia, the greatest excesses being for acute myeloid leukaemia and the other lymphatic cancers. The present study shows no excess mortality from all cancers or all leukaemias combined, and the data for acute myeloid leukaemia and other lymphatic cancers are inconclusive. The findings of the present study should be seen in the context of the choice of study sample which included everyone resident near all identified electricity transmission installations in defined areas; with the result that the majority of the sample were resident next to relatively low current sub-stations. The study's inability to confirm the major associations suggested by earlier work may, if such associations are genuine, be due to the low exposure levels probably experienced by most of the study sample, to dilution of exposure by movement to and from study houses or to other inadequacies of the study design. The mortality of the study sample will continue to be followed increasing the number of cases available for analysis. However, the evidence outlined in Table VII suggests that further research is needed to clarify the effects, if any, of exposure to higher levels of electro-magnetic fields.

**Table VII** Summary of evidence for association between electro-magnetic radiation and (i) all cancers, (ii) leukaemias and (iii) other lymphatic cancer etc.

| | | *All cancers* | | *All leukaemias ICD (9) 204–208* | | *Lymphoid leukaemias (204)* | | *All acute leukaemias (204.0, 205.0, 206.0, 207.0, 208.0)* | | *Acute myeloid leukaemias (205.0)* | | *Other lymphatic cancers etc. (200–203)* | |
|---|---|---|---|---|---|---|---|---|---|---|---|---|---|
| *Study* | *Cases* | *O* | *E* | *O* | *E* | *O* | *E* | *O* | *E* | *O* | *E* | *O* | *E* |
| Wertheimer & Leeper (1979) | Children resident near high currrent configurations mortality | 129 | 74.0 | 63 | 29.0 | — | — | — | — | — | — | 18 | 11 |
| Fulton *et al.*, (1980) | Children resident with 'high' and 'very high' exposure incidence | — | — | 103 | 99 | — | — | 86 | 86.5 | 25[a] | 23.5[a] | — | — |
| Tomenius *et al.* (1982) | Children resident near high voltage wire incidence | 32 | 15.1 | — | — | — | — | — | — | — | — | — | — |
| Wertheimer & Leeper (1982) | Adults resident near high and very high current configurations mortality and survivors | 438 | 372 | | | (Significant association with lymphomas noted – no data given) | | | | | | | |
| Milham (1982) | Electrical workers mortality | — | — | 136 | 99.2 | — | — | 60 | 36.7 | — | — | — | — |
| Wright *et al.* (1982) | Electrical workers incidence | — | — | 35 | 27.2 | — | — | 23 | 13.3 | 22 | 10.6 | — | — |
| McDowall (1983) | Electrical workers mortality 1970–72 | — | — | 85 | 86.7 | 28 | 28 | — | — | 31 | 29.8 | — | — |
| | mortality 1973 | — | — | — | — | — | — | — | — | 36 | 14.5 | — | — |
| Coleman *et al.*, (1982) | Electrical workers incidence | — | — | 113 | 96.6 | 45 | 33.8 | — | — | 33 | 26.8 | — | — |
| Milham (1982*a, b*) | Amateur radio operators mortality | 451 | 372.8 | 24 | 12.6 | 3 | 3.9 | — | — | 11 | 3.8 | 39 | 19.8 |
| Pearce *et al.* (1985) | Electrical workers incidence | — | — | 18 | 10.8 | — | — | — | — | 4 | 3.4 | — | — |
| Present study | Persons resident near electricity transmission facilities mortality | 213 | 213 | 6 | 5.9 | — | — | — | — | 3 | 2.1 | 10 | 7.8 |
| | (resident within 15 metres) | (27 | 26.2) | (1 | 0.7) | — | — | — | — | (— | 0.3) | (3 | 0.9) |

[a]Includes all acute leukaemias less acute lymphoid leukaemia; O—Observed deaths; E—Expected deaths.

### References

ADELSTEIN, A. & MARDEN, C. (1975). Suicides 1961–1974, *Population Trends*, **2**, 13.

ALDRICH, T.E., GLORIEUX, A. & CASTRO, S. (1984). Florida cluster of five children with endodermal sinus tumours. *Oncology*, **41**, 233.

BONNELL, J.A. (1982). Effects of electric fields near power-transmission plant. *J. Royal Soc. Med.*, **75**, 933.

BRYAN, F.A. (1980). Re: *Electrical Wiring Configurations and Childhood Leukaemia in Rhode Island. Am. J. Epidemiol.*, **112**, 419.

COLEMAN, M., BELL, J. & SKEET, R. (1983). Leukaemia incidence in electrical workers. *Lancet*, **i**, 982.

EASTERLY, C.E. (1981). Cancer link to magnetic field exposure: A hypothesis. *Am. J. Epidemiol.*, **114**, 169.

EDITORIAL, (1983). Electromagnetism and cancer. *Lancet*, **i**, 224.

ELECTRICITY COUNCIL, (1985). Handbook of electricity supply statistics 1984, Electricity Council, London.

FULTON, J.P., COBB, S., PREBLE, L., LEONE, L. & FORMAN, E. (1980). Electrical wiring configurations and childhood leukaemia in Rhode Island. *Am. J. Epidemiol.*, **111**, 292.

HAMPT, R.C. & NOLFI, V.R. (1984). The effects of high voltage transmission lines on the health of adjacent resident populations. *Am. J. Public Health*, **74**, 76.

LIN, R.S., DISCHINGER, P.C., CONDE, J. & FAWELL, K.P. (1985). Occupational exposure to electro-magnetic fields and the occurrence of brain tumours. *J. Occ. Med.*, **27**, 413.

McDOWALL, M. (1983). Leukaemia mortality in electrical workers in England and Wales. *Lancet*, **i**, 246.

McDOWALL, M. (1984). 'Reference rates from routine statistics' in expected numbers in cohort studies. MRC. Environmental Epidemiology Unit. *Scientific Report*, **No. 6**, MRC Southampton General Hospital.

MILD, K.H. & OBERG, P.A. (1982). Neurophysiological effects of electromagnetic fields – A critical review. *Electroencephalo. Clin. Neurophysiol.* Supp: **36**, 715.

MILHAM, S. (1982). Mortality from leukaemia in workers exposed to electrical and magnetic fields. *N. Engl. J. Med.*, **307**, 269.

MILHAM, S. (1985*a*). Silent keys: Leukaemia mortality in amateur radio operators. *Lancet*, **i**, 812.

MILHAM, S. (1985*b*). Personal communication.

MILLER, M.W. (1980). Re: *Electrical Wiring Configurations and Childhood Cancer. Am. J. Epidemiol.*, **112**, 165.

OPCS (1970). Classification of occupations 1970. HMSO: London.

OPCS (1978). Occupational mortality 1970–72. HMSO: London.

OPCS (1981). Area mortality decennial supplement 1969–73. Series DS. No. 4. HMSO: London.

PEARCE, N.E., SHEPPARD, R.A., HOWARD, J.K., FRASER, J. & LILLEY, B.M. (1985). Leukaemia in electrical workers in New Zealand. *Lancet*, **i**, 811.

PERRY, F.S., REICHMANIS, M., MARINO, A.A. & BECKER, R.O. (1981). Environmental power frequency – Magnetic fields and suicide. *Health Physics*, **41**, 267.

REICHMANIS, M., PERRY, F.S., MARINO, A.A. & BECKER, R.O. (1979). Relation between suicide and the electromagnetic field of overhead power lines. *Physio. Chem. Phys.*, **11**, 395.

SAINSBURY, P. & JENKINS, J.S. (1982). The accuracy of officially reported suicide statistics for purposes of epidemiological research. *J. Epidemiol. Comm. Health.*, **36**, 43.

TOMENIUS, L., HELLSTROM, L. & ENANDER, B. (1982). Electrical constructions and 50 HZ magnetic field at the dwellings of tumour cases (0–18 years of age) in the county of Stockholm. Abstract: *International Symposium on Occupational Health and Safety in Mining and Tunnelling, Prague.*

WERTHEIMER, N. & LEEPER, E. (1979). Electrical wiring configurations and childhood cancer. *Am. J. Epidemiol.*, **109**, 273.

WERTHEIMER, N. & LEEPER, E. (1980). Re. *Electrical wiring Configurations and Childhood Leukaemia in Rhode Island. Am. J. Epidemiol.*, **11**, 461.

WERTHEIMER, N. & LEEPER, E. (1982). Adult cancer related to electrical wires near the home. *Int. J. Epidemiol.*, **11**, 345.

WRIGHT, W.E., PETERS, J.M. & MACK, T.M. (1982). Leukaemia in workers exposed to electrical and magnetic fields. *Lancet*, **ii**, 1160.